# Dietary Habits and Obesity in Middle-Aged and Elderly Europeans—The Survey of Health, Ageing, and Retirement in Europe (SHARE)

**DOI:** 10.3390/nu17152525

**Published:** 2025-07-31

**Authors:** Manuela Maltarić, Jasenka Gajdoš Kljusurić, Mirela Kolak, Šime Smolić, Branko Kolarić, Darija Vranešić Bender

**Affiliations:** 1 Andrija Štampar Teaching Institute of Public Health, Mirogojska 16, 10000 Zagreb, Croatia; manuela.maltaric@stampar.hr (M.M.); branko.kolaric@stampar.hr (B.K.); 2 Faculty of Food Technology and Biotechnology, University of Zagreb, Pierottijeva 6, 10000 Zagreb, Croatia; dvranesci@kbc-zagreb.hr; 3 School of Medicine, University of Zagreb, Šalata 3, 10000 Zagreb, Croatia; mirela.kolak@student.mef.hr; 4 Faculty of Economics and Business, University of Zagreb, Trg J.F. Kennedy 6, 10000 Zagreb, Croatia; ssmolic@efzg.hr; 5 Unit of Clinical Nutrition, Department of Internal Medicine, University Hospital Centre Zagreb, Kišpatićeva 12, 10000 Zagreb, Croatia

**Keywords:** mediterranean diet adherence, ageing, obesity, handgrip strength, SHARE

## Abstract

**Background/Objectives**: Understanding the impact of dietary habits in terms of obesity, health outcomes, and functional decline is critical in Europe’s growing elderly population. This study analyzed trends in Mediterranean diet (MD) adherence, obesity prevalence, and grip strength among middle-aged and elderly Europeans using data from the Survey of Health, Ageing and Retirement in Europe (SHARE). **Methods**: Data from four SHARE waves (2015–2022) across 28 countries were analyzed. Dietary patterns were assessed through food frequency questionnaires classifying participants as MD-adherent or non-adherent where adherent implies daily consumption of fruits and vegetables and occasional (3–6 times/week) intake of eggs, beans, legumes, meat, fish, or poultry (an unvalidated definition of the MD pattern). Handgrip strength, a biomarker of functional capacity, was categorized into low, medium, and high groups. Body mass index (BMI), self-perceived health (SPHUS), chronic disease prevalence, and CASP-12 scores (control, autonomy, self-realization, and pleasure evaluated on the 12-item version) were also evaluated. Statistical analyses included descriptive methods, logistic regressions, and multiple imputations to address missing data. **Results**: A significant majority (74–77%) consumed fruits and vegetables daily, which is consistent with MD principles; however, the high daily intake of dairy products (>50%) indicates limited adherence to the MD, which advocates for moderate consumption of dairy products. Logistic regression indicated that individuals with two or more chronic diseases were more likely to follow the MD (odds ratio [OR] = 1.21, confidence interval [CI] = 1.11–1.32), as were those individuals who rated their SPHUS as very good/excellent ([OR] = 1.42, [CI] = 1.20–1.69). Medium and high maximal handgrip were also strongly and consistently associated with higher odds of MD adherence (Medium: [OR] = 1.44, [CI] = 1.18–1.74; High: [OR] = 1.27, [CI] = 1.10–1.48). **Conclusions**: The findings suggest that middle-aged and older adults are more likely to adhere to the MD dietary pattern if they have more than two chronic diseases, are physically active, and have a medium or high handgrip. Although an unvalidated definition of the MD dietary pattern was used, the results highlight the importance of implementing targeted dietary strategies for middle-aged and elderly adults.

## 1. Introduction

The world population is ageing, and it is projected that the twenty-seven European Union countries (EU-27) will have close to half a million centenarians by 2050 [1]; therefore, this issue is also a focus for the public sector. Disease prevention and maintaining good physical and cognitive abilities will certainly contribute to the general quality of life of older people.

Over half of older adults live with at least one chronic illness, as ageing significantly increases the risk of various health conditions. For instance, cardiovascular diseases (CVDs) affect up to 70% of the elderly, while diabetes is present in 20% of this population [2,3,4,5]. CVDs are the leading cause of death and illness among adults and the elderly worldwide, primarily driven by both coronary and non-coronary atherosclerosis [6,7,8]. Plaque instability due to chronic inflammation increases the risk of rupture, thrombosis, and acute cardiovascular events such as myocardial infarction or stroke [9]. Risk factors such as hypertension, diabetes, hyperlipidemia with high low-density lipoprotein (LDL) and low high-density lipoprotein cholesterol (HDL), and smoking exacerbate disease progression, highlighting the importance of preventive and anti-inflammatory strategies [10]. Common cardiovascular conditions in older adults include coronary artery disease, heart failure, valvular heart disease, and atrial fibrillation [11,12,13]. Non-pharmacological approaches, including tailored lifestyle modifications, and comprehensive management of comorbidities, are essential for optimizing overall health outcomes [14,15]. The buildup of excess lipids in the body, seen in conditions such as hyperlipidemia and increased visceral fat, is associated with various chronic lipid-related diseases, such as CVD, type 2 diabetes, and obesity [16]. Managing dyslipidemia in elderly patients involves individualized treatment plans that consider overall health, comorbidities, and life expectancy. Statin therapy has been shown to reduce CVD events in this population, but potential benefits must be weighed against risks such as muscle and hepatic toxicity, especially in patients with multiple comorbidities or advanced age. Obesity can speed up the ageing process and increase the risk of numerous age-related conditions, including sarcopenia, type 2 diabetes, CVD, and various lipid-related disorders, posing a significant threat to the health of the elderly [17] as well as loss of bone density and sarcopenia [18,19,20]. Therefore, it is necessary to monitor the general condition of the elderly population, and biomarkers that would indicate their health status are being investigated, and handgrip strength has been singled out as one of the essential biomarkers. Research shows a correlation between handgrip strength and the strength of other muscle actions in healthy individuals and those with certain pathologies [21], such as metabolic syndrome which arises from a complex interplay of genetic, environmental, and lifestyle factors, including unhealthy dietary habits, physical inactivity, and excess body weight. Its global prevalence is on the rise, largely due to the growing incidence of obesity—particularly abdominal obesity—and type 2 diabetes. Key physiological mechanisms driving this trend include heightened insulin resistance, ageing, and central fat accumulation, all of which contribute to the syndrome’s increasing occurrence [21,22]. Managing metabolic syndrome primarily requires lifestyle modifications. Key components of treatment include weight reduction, a balanced diet, and regular physical activity. Addressing modifiable risk factors, such as hypertension and dyslipidemia, should follow established medical guidelines. Additionally, early diagnosis and timely intervention play a vital role in reducing the likelihood of related cardiovascular complications [23,24,25]. The Mediterranean diet (MD) abundant in fruits, vegetables, whole grains, legumes, nuts, and olive oil, has been associated with improved blood pressure, lipid profiles, and insulin sensitivity, thereby reducing the risk of CVDs, obesity, sarcopenia and bone density [26] and is beneficial for managing metabolic syndrome, glucose, and lipid dysregulation due to its emphasis on anti-inflammatory and antioxidant-rich foods. A meta-analysis found that high adherence to the MD reduces all-cause mortality risk by 23%, cardiovascular mortality by 27%, and non-fatal cardiovascular events by 23% [27]. This dietary pattern, Adherence to the MD has been linked to reductions in central obesity and insulin resistance, key components of metabolic syndrome. Additionally, this diet lowers triglyceride levels and low-density lipoprotein cholesterol while raising high-density lipoprotein cholesterol, enhancing lipid profiles. Furthermore, the MD helps regulate blood glucose levels, reducing the likelihood of developing type 2 diabetes [28].

Large-scale health surveys such as the Survey of Health, Ageing, and Retirement in Europe (SHARE) project collect different data for the population over 50 [29,30], which in its latest wave (wave 9, data collected in 2021/22) includes data from 28 countries, including Croatia (since 2015).

Given the lack of studies that investigate the relationship between eating habits, nutritional status, physical (in)activity, and biomarkers of health status (such as maximum hand grip strength) in the middle-aged and elderly population, the aim of our study is to investigate the potential relationships of these parameters. The goal is to investigate the above through large-scale studies, such as the SHARE study. Therefore, we analyzed and investigated the trends of changes in the eating habits of the elderly population in Europe in the last 10 years, including 28 countries, their nutritional status (potential obesity) as well as their handgrip strength as an indispensable biomarker in the elderly. For the first time, data from four waves in the SHARE database (wave 6, wave 7, wave 8, and wave 9) were presented.

## 2. Materials and Methods

### 2.1. Study Population

**Figure 1:**
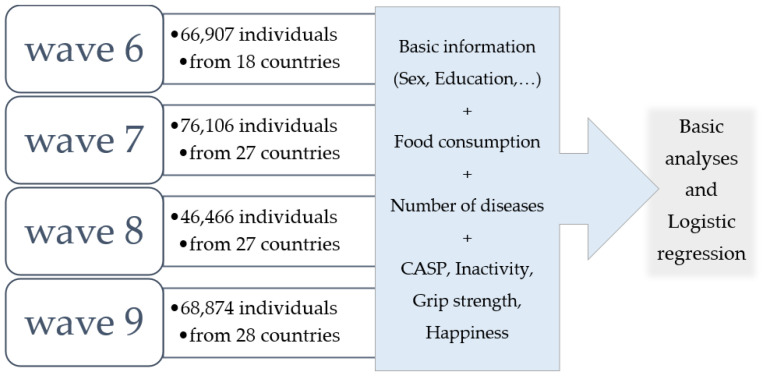
Data profile used in this study.

The presented study included data from waves 6, 7, 8, and 9 of the Survey of Health, Ageing and Retirement in Europe (SHARE), a survey recruiting individuals aged 50 years or older residing in European countries and Israel using a panel methodology [29,30]. Participants older than 51 were included in the study. Figure 1 provides more details about the study sample. The SHARE study′s target demographic consists of all people who are at least 50 years old, speak the nation′s official language, do not reside overseas or in a facility such as a prison, are married or have a partner. Households with at least one member meeting the aforementioned requirements are also considered to be part of the target population. Additional criteria in our study were the availability of anthropometric data, education level, number of illnesses, handgrip strength, and answers to questions about dietary habits and physical activity. All other subjects for whom the aforementioned criteria were unavailable were excluded from the analyses.

SHARE is an extensive longitudinal project that gathers cross-national microdata across multiple disciplines, focusing on health, financial well-being, and social and familial dynamics among older adults. Croatia has contributed to the SHARE initiative since 2004. The survey features nationally representative samples of individuals aged 50 and above from each participating European nation and Israel. Information is collected via in-person interviews using the Computer-Assisted Personal Interviewing (CAPI) method [31,32,33]. This study assessed SHARE data associated with the Mediterranean pattern and maximum handgrip strength across four distinct cycles, with a focus on tracking changes in trends over time. The SHARE data analyzed in this study are presented in Figure 1.

### 2.2. Mediterranean Diet Patterns (Note: Unvalidated Definition of MD Pattern)

A modified food frequency questionnaire was used for data collection [33]. It includes a series of questions designed to evaluate the quality of participants′ dietary habits. Dietary quality was measured through the following items:

- How frequently do you consume a serving of dairy products (e.g., a cup of yoghurt, cheese, a glass of milk, or a can of high-protein supplements) each week?
- How often do you eat a serving of eggs, beans, or legumes per week?
- How frequently do you consume a serving of poultry, meat, or fish on a weekly basis?
- How often do you eat a serving of fruits and vegetables each week?

Participants responded using a five-point scale with the following options: (i) less than once a week; (ii) once a week; (iii) twice a week; (iv) 3 to 6 times a week, and (v) every day [27,28,29,30].

By incorporating data from 27 European countries and Israel, the core objective remained consistent: promoting the health and well-being of middle-aged and older adults by associating them with the benefits of the MD pattern. Previously mentioned modified calculations of the MD pattern include regular consumption of fruits and vegetables, and frequent intake of eggs, beans, legumes, meat, fish, or poultry (three to six times per week) (4,6). From the frequency data of these food groups (excluding dairy), we constructed a binary index to classify participants as adherent or non-adherent to the Mediterranean diet (1 = adherent, 0 = non-adherent). A limitation of the SHARE database is that it does not differentiate between red meat and other meats [31,32,33,34]; our results were compared with studies acknowledging this limitation and results should be interpreted with caution because an unvalidated definition of MD was used.

### 2.3. Biomarker–Grip Strength

Handgrip strength, i.e., the average maximum static handgrip force, measured using a dynamometer, is a predictor of numerous future outcomes. It is a simple and inexpensive method that provides insights into and limitations of general physical health [35]. Available data on the maximal grip strength (from the SHARE study) were divided into three classes according to sex: (i) low, values 1–25% of the maximum; (ii) medium, 26–50%, and (iii) high, over 50%.

### 2.4. General Health Indicators

Health was monitored based on the degree of nutrition, i.e., body mass index (BMI). Two scales were used to monitor normal nutrition: a scale for people under 65 years of age [36] and the European Society for Clinical Nutrition and Metabolism (ESPEN) recommendations for people over 65 years of age [37]. Normal nutritional status is expected to be 18.5–24.99 kg/m^2^ for people under 65, while the expected range for those over 65 is 21–27.49 kg/m^2^ [36,37]. The second parameter that indicates the health status of the subjects is the number of chronic diseases available in the database (under the variable “cronic2”). Two categories are distinguished: (i) less than two chronic diseases and (ii) two or more chronic diseases [38]. The query associated with chronic diseases was used to gain insight into the potential connection between the number of diseases and the increase in followers of the Mediterranean diet. The third indicator associated with health is the parameter “self-perceived health” (SPHUS-1) in which respondents had the option of choosing from three categories: Poor/Fair, Good or Very good, and Excellent. The fourth indicator, SPHUS-2, is similar to the previously mentioned, but has only two categories (very good/excellent and less than very good).

### 2.5. CASP-12 Index

Control, autonomy, self-realization, and pleasure, evaluated using the 12-item version (CASP-12), are indicators of quality of life in older age [31,38]. CASP-12 consists of 12 questions that are evaluated according to the Likert scale (“often”, “sometimes”, “rarely”, “never”). The expected range for CASP-12 is a minimum of 12 points to a maximum of 48 points. Based on the study by Maltarić et al. [32], three categories were created: low (12–24 points), medium (25–36 points), and high (more than 36 points).

### 2.6. Covariate Variables

Covariates in the analysis included: Age (range: 51–104 years), Sex (Male/Female), Marital status (“living with partner” vs. “not living with partner”), Years of education (<9 years: primary, 9–12 years: secondary, >12 years: tertiary), Current job situation (retired vs. not retired), Self-perceived health (SPHUS-1: Poor, Fair, Good, Very good, and Excellent; SPHUS-2: Very good/excellent and Less than very good), Quality of life (CASP-12: Low, Medium, High), Physical inactivity (Never vigorous nor moderate physical activity vs. other), Chronic medical diagnoses (less than 2 (<2) vs. 2+), and BMI (normal by age group).

### 2.7. Statistical Analysis

Before data processing, missing data were analyzed by country and observed parameters. According to prior research, analysis is reliable if missing values are below 5%, which was not the case for some variables (e.g., education level and health indicators). Therefore, multiple imputation was used to increase data completeness, following the method in [39], where it is suggested to investigate the observed parameter with and without missing data by use of a *t*-test. If the test shows no statistically significant differences (*p* < 0.05), the data can be taken into observation. The *p*-values for education levels (wave 6 to wave 9) were in the range of 0.09–0.45, while for the health indicators (max. handgrip) the *p*-value ranged from 0.12 to 0.31. The SHARE dataset includes coded nominal and scale variables [38]. Depending on the data type, appropriate tests were applied to compare individuals who followed the MD with those who did not, and those with low, medium, or high maximal grip. Categorical variables were expressed as percentages. After confirming normality using the Shapiro–Wilk test, continuous variables were presented as means and standard deviations (SDs). Comparisons between groups were conducted using a two-sided paired *t*-test. To account for sex differences and meaningful results, handgrip strength was categorized into sex-stratified tertiles [40]. Additional analyses also accounted for handgrip strength as a continuous variable [41].

Logistic regression was used to analyze the relationship of Mediterranean diet adherence with the number of chronic diseases, body mass index, CASP, physical inactivity, SPHUS, and max. handgrip strength. Three models were developed: (i) a crude model (in which no adjustment was made for confounding factors), then (ii) Model 1 (in which adjustment was made for age, sex and marital status), and (iii) Model 2 (which was additionally adjusted for level of education and current job status). All statistical analyses were performed using SPSS v. 19 (version 19.0, SPSS Inc., Chicago, IL, USA).

## 3. Results

**Figure 2:**
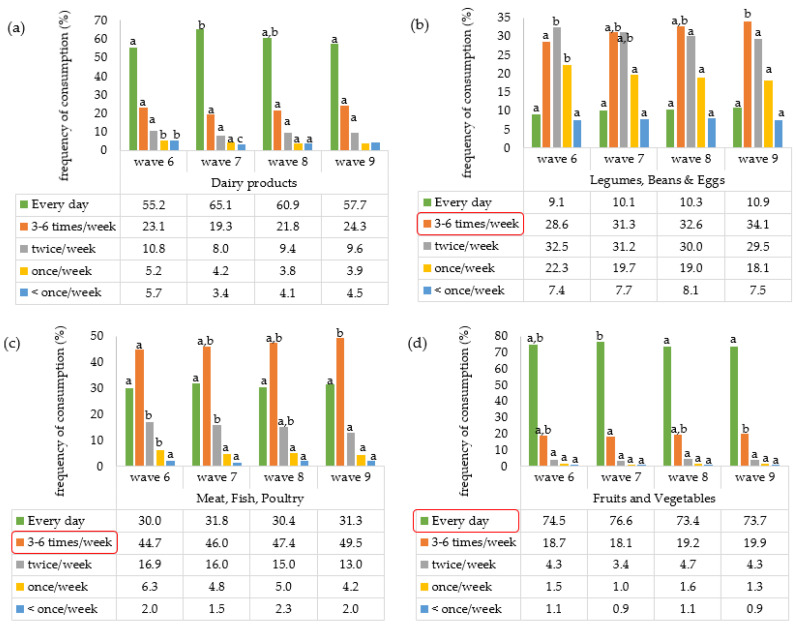
Frequency of average weekly consumption of: (**a**) dairy products, (**b**) legumes, beans and eggs, (**c**) meat, fish and poultry, (**d**) fruits and vegetables. The red box indicates the frequency of consumption of the listed food groups based on which the Mediterranean dietary pattern (MD) was calculated. Wave 6, data from 2015; wave 7, data from 2017; wave 8, data from 2019/20; wave 9, data from 2021/22. Different letters for the same food consumption frequency (same colour) indicate significant differences in consumption in different waves. The consumption frequencies used in assessing adherence to the Mediterranean diet are highlighted with a red frame.

Four waves of data collection are included in this study (w6: 2015; w7: 2017; w8: 2019/20; w9: 2021/22). The data were analyzed separately for each wave. The first step was the presentation of frequencies of food consumption based on four different food groups: (i) dairy products; (ii) eggs, beans, or legumes; (iii) poultry, meat, or fish; and (iv) fruits and vegetables (Figure 2). The figure indicates significant differences in the frequencies (%) of average weekly consumption of foods from these four main food groups (*p* < 0.05).

High daily intake of dairy products (>50% of respondents in every wave) may indicate limited adherence to the Mediterranean dietary pattern (MD), which advocates moderate consumption of dairy. In contrast, the consumption pattern of legumes, beans, and eggs aligns more closely with MD recommendations, emphasizing moderation rather than daily intake. Notably, the MD-aligned frequency category (3–6 times/week) represents the largest share of respondents (31–35%) across waves. For meat, fish, and poultry, the 3–6 times/week category is similarly dominant (44–50%), making it the most MD-compatible group in terms of frequency. However, this pattern only partially aligns with MD principles, which recommend limited red meat intake and moderate fish consumption. Finally, a very high proportion of respondents report daily consumption of fruits and vegetables (74–77%), a pattern that remained stable over time. This suggests strong alignment with MD guidelines, which encourage daily intake, despite the use of the 3–6 times/week category as the operational criterion for MD adherence in this analysis.

Average values of the basic participant characteristics from the available datasets from waves 6 to 9 are presented in Table 1, where the Mediterranean diet pattern (MD) and grip strengths per observed variables were investigated. Table A1 and Table A2 contain values for each of the 28 included countries, for the followers of MD (Table A1) and Maximal grip strength (Table A2), per waves of data collection (w6–w9).

Table 1 indicates the higher number of women in the population, and also highlights the proportion with a normal BMI in the population older than 65, which is a direct consequence of the implementation of ESPEN recommendations. Interestingly, a significant proportion of older adults with medium and high handgrip strength are living with a partner (68.8% (medium) and 75.2% (high) compared with 31.2% (medium) and 24.6% (high) for people without partners). The proportion of people with two or more chronic diseases with a weak handgrip is exceptionally high (62.7%), and the proportion of those following the MD among them is also high (53.8%). In terms of self-perceived health, participants who rated their health as good included 37.9% of those following the MD pattern, and 40.8% and 41.4% of those with medium and high handgrip.

BMI was used as one of the health indicators, and its frequencies are shown in Table 2. The trends in all available data from the four waves (w6–w9) by country are shown in Supplementary Tables S1 and S2. In the Supplementary Tables, heat maps were used to make visualizing the similarities and/or differences in trends easier. Two BMI categorizations are used in Table 2: the standard categories for adults and the ESPEN recommendation for assessing the nutritional status of individuals over 65 [37].

**Figure 3:**
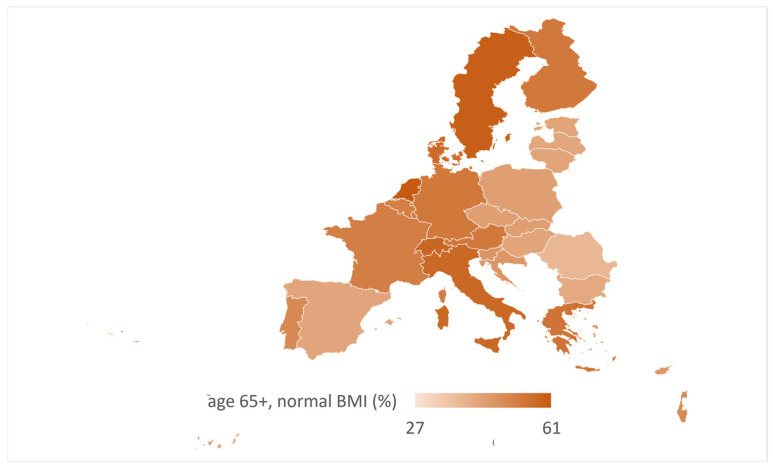
The proportion of adults participating in the SHARE study, aged over 65 with a normal BMI (BMI in the range of 21–27.49 kg/m^2^), based on data from wave 9 (data collected in 2021/22).

The distribution of the proportion of people over 65 with a normal BMI based on data from the last published wave (wave 9) is shown in Figure 3.

The lowest proportion of people over the age of 65 with a normal BMI is in Malta (26.8%), followed by Romania (38.2%); countries in orange have 40–50% of their over-65-year-old age group with a normal range; and countries in blue are those where around 60% of respondents have a BMI in the normal range (Sweden with 59.9% and The Netherlands with 61.1%). However, it is worth noting the Supplementary Tables, which show that Malta had the highest number of respondents with a BMI < 21 kg/m^2^ (26.3%). There is also a visual difference in the distribution trends in BMI categories for people under 65. In the above heat maps, blue indicates the highest value in the same wave, while red indicates the lowest. The distribution is extremely varied when looking at the BMI of people under 65. For example, Croatia has among the lowest numbers of people whose BMI is normal (only 28.3%), and the proportion of people with excess body weight and obesity is more than 68%. Meanwhile, the heat map for the proportion of BMI for people over 65 shows a clear dominance of the proportion of people with a normal BMI. However, the “underweight” and “obese” groups are also pronounced, which, along with age, can result in several complications if the person suffers from one or more chronic diseases. Croatia’s participation in the SHARE study is important for both research and policymaking. It allows for the collection of comparable data on the health and socioeconomic situation of older adults, which is essential for planning public policies for an ageing society. As one of the ten oldest countries in the European Union, Croatia faces serious societal challenges that require reliable, long-term data. The cross-national perspective of SHARE helps place Croatia’s experience in a wider European context and supports the identification of useful policy solutions.

The two observed binary outcomes were (i) following the Mediterranean dietary pattern (yes = 1; no = 0) and (ii) low grip strength (Low = 1; Other = 0). Those results are presented in Table 3. Odds ratios (ORs) with 95% confidence intervals (CIs) were calculated. If the CI includes 1, then the OR estimate is not statistically significant at the chosen confidence level (usually 95%). The true OR in the population may be less than 1, greater than 1, or exactly 1. If the CI does not include 1, then the OR estimate is statistically significant, i.e., it can be said with 95% confidence that the true OR in the population lies within that range and is not 1. When the entire interval is above 1, there is a significant increase in the odds, and if the entire interval is below 1, there is a significant decrease in the odds. The adjustment included age, sex, marital status, education level, and current job status of participants in the SHARE project wave 9. Bolded values indicate significant relationships; for example, a higher number (but not significant) of respondents who follow the principles of the MD can be seen if they have been diagnosed with two or more chronic diseases, i.e., if the respondent has two or more diagnosed chronic diseases, the probability of following the principles of the Mediterranean diet increases by 1.23 times.

The results of the models of logistic regression, which were used to investigate the association of quality of life parameters and MD adherence, are presented for three models using odds ratios (ORs) and *p*-values. In the middle-aged and elderly population, having 2 or more chronic diseases significantly increases the odds (ORs: 0.07 to 0.19, all *p* < 0.001) of following the MD pattern. For the BMI, normal BMI shows a modest but significant association in the Model 3 (*p* = 0.048), but overweight and obesity show weaker or non-significant effects. CASP (Quality of Life): Medium CASP scores are significantly associated with reduced odds in Models 1 and 2 (*p* < 0.05), but not in Model 3. High CASP loses significance across models. Physical activity (defined as “Other”) which excludes “never engaging in physical activity” significantly increases odds of following the MD pattern across all models (*p* < 0.001). Very good and excellent elf-perceived health (SPHUS) ratings are strongly and consistently associated with higher odds (*p* < 0.01). Although the grip strength differs by sex, regardless the models (*p* < 0.005), both medium and high grip strength are significantly associated with increased odds across all models.

## 4. Discussion

The study investigated the complex relationships of the health and nutritional status of middle-aged and elderly citizens of the EU countries. The ability to monitor trends (potential changes) over a longer period of time is possible thanks to long-term studies, such as SHARE. Dietary patterns and their impact on health are the focus of many studies and should certainly be highlighted. Table A1 shows an increase in the proportion of middle-aged and older people who adopt the Mediterranean dietary pattern.

There is a clear connection between the MD and health. The NU-AGE project investigated the impact of the MD on the gut microbiome in older people and found that it resulted in reduced frailty and improved health status in five European countries: the UK, France, the Netherlands, Italy, and Poland [42,43,44]. We emphasize that our study used an extremely simplified method of assessing the Mediterranean dietary pattern using the SHARE study data (unvalidated definition of MD pattern), which is also available in other publications [32,33,34,45], but the best MD adherence indicator is the scoring system, listed among the most common used ones, in Table A3. Publications resulting from the SHARE study open new avenues for discussions and point to new important questions; for example, in the latest version (wave 9), a question was introduced (for the first time) on whether the respondents were vegetarians. To minimize misinterpretations of the results, people who declared themselves vegetarians in wave 9 were not included in the calculation of the Mediterranean dietary pattern.

The findings of this study align with a growing body of evidence underscoring the pivotal role of dietary patterns in influencing obesity and physical function in middle-aged and older adults in Europe. Recent longitudinal analyses have highlighted a positive trend in adherence to the MD across various European populations. For instance, a study utilizing SHARE data from 2013 to 2019 observed a significant increase in MD adherence, particularly among younger, educated, and higher-income groups [46]. Adherence to the MD has been consistently associated with favourable body composition outcomes. The Lookup 7+ project demonstrated that older adults with higher MD adherence exhibited lower relative fat mass, suggesting a protective effect against adiposity [47]. These findings are corroborated by a comprehensive study which found that individuals maintaining a diet rich in fruits, vegetables, whole grains, and healthy fats had a higher likelihood of achieving “healthy ageing”, defined as reaching 70 years without major chronic diseases and with preserved cognitive and physical function. Beyond body composition, dietary patterns also influence muscle strength and function. The LifeAge study found that adherence to the MD, combined with regular physical activity, was associated with a lower prevalence of sarcopenia among European older adults [48]. Similarly, a pooled analysis of four longitudinal ageing cohorts revealed that higher protein intake, especially when coupled with physical activity, attenuated the decline in grip strength over time [49]. Potential recall bias in the food consumption questionnaire should be highlighted as a limitation of the presented MD adherence; however, as the questionnaires are designed to estimate people′s usual eating patterns [50], it should be emphasized that the individuals collecting the data included in the SHARE study were trained (using the same programme in the different countries) to help the respondents to provide the most credible answers. The diversity of dietary patterns in the EU is also undeniable, and according to the study by Maltarić et al. [32], which analyzed the data collected in wave 8, some statistically significant differences were identified, including the dominance of meat, fish, and poultry consumption 3–6 times each in Central and Eastern Europe (43.5%), Northern Europe (55.5%) and Western Europe (52.8%), while 46.9% of respondents from Southern European countries declared that they consumed foods from the aforementioned group daily. Unfortunately, these data do not show the prevalence of red meat compared with white meat and/or fish, which would be clear using common indices (Table A3) [50,51,52,53,54]. This is an indicator of how the number of queries associated with the dietary habits of respondents could be expanded within the SHARE study.

Conversely, diets high in ultra-processed foods have been linked to adverse outcomes. A prospective analysis from the TCLSIH study indicated that increased consumption of ultra-processed foods was associated with a more pronounced decline in grip strength among middle-aged and older adults [55]. This underscores the importance of dietary quality in preserving musculoskeletal health. Furthermore, the interplay between diet and inflammation has garnered attention. An anti-inflammatory dietary pattern has been associated with a slower decline in handgrip strength, suggesting that reducing dietary inflammation may be beneficial for maintaining muscle function in older age [56].

These insights collectively emphasize the multifaceted impact of diet on ageing. Promoting dietary patterns emphasizing whole, minimally processed foods, adequate protein intake, and anti-inflammatory properties may effectively combat obesity and preserve physical function among Europe’s ageing population. Future public health initiatives should consider these dietary factors to enhance healthy ageing trajectories. Physical activity, which was included in this study under the parameter “physical inactivity”, was unfortunately not discussed further, but represents a significant variable of good health and will be the subject of a study focusing on a smaller dataset (selected respondents from Croatia). It is extremely important to take care of bone quality, muscle mass, and potential sarcopenia in the elderly population; MD and physical activity that positively affected these parameters [57,58,59].

Following the irreplaceable biomarker for the elderly population [21], Denmark and Slovenia (>10%) have the highest average share of the population (in the last 10 years), whose pressure is in the “high” category, according to the data in Table 2, while the share of the population whose pressure is the highest in the “Low” category is Spain (wave 9 = 39.6%), and Cyprus (40.8%). Low values of handgrip are associated with a higher risk of mortality from CVDs and cancer, and a greater risk of chronic diseases as well as malnutrition, fractures, etc. [35,42].

A weak handgrip may indicate decreased muscle mass and strength (sarcopenia), which is a common problem in older adults [60]. Sarcopenia has been associated with an increased risk of falls, fractures, disability, and mortality [18]. When considering functional ability and handgrip strength as a predictor, one refers to the need for strength in the hands for many everyday activities, such as carrying objects, opening jars, standing up from a chair, and performing personal hygiene activities. A weak handgrip can make these activities more difficult and lead to reduced independence and quality of life [18,60]. Handgrip strength as a biomarker of health status has been confirmed by a number of studies that have linked a weak grip with an increased risk of various chronic diseases, including CVDs, type 2 diabetes, chronic obstructive pulmonary disease (COPD), kidney failure, dementia and cognitive impairment, depression, and malnutrition, and an increased risk of postoperative complications and longer recovery. Numerous studies have shown that a weak handgrip is a strong and independent predictor of premature mortality in older adults, even when other risk factors are considered. This is due to muscle physiology, hormonal changes, and nutritional status, which change with age. There is a natural loss of muscle fibres, especially fast-twitch type II fibres that are important for strength (fast, glycolytic, white), with age. There is also a decrease in neuromuscular efficiency [18,19,20,60].

However, the relationship between handgrip strength and BMI in older adults is complex and sometimes contradictory. What is worrying is the proportion of people aged 50–64.9 who are overweight (men = 45.7% and women = 34.8%), while the proportion of obese people is almost a quarter (men = 26.5% and women = 24.4%). Some studies show an inverse relationship, where a higher BMI may be associated with weaker handgrip strength, probably due to a greater amount of fat tissue relative to muscle mass [27,61,62]. Other studies suggest that a moderately higher BMI may be associated with a stronger grip, possibly due to a higher total body mass [27]. Therefore, it is important to note that the quality of muscle mass, and not just total body mass, is crucial for grip strength and general health. Older people with high BMI but low muscle mass (sarcopenic obesity) may still have poor hand grip and an increased risk of negative health outcomes. Recent research suggests that normalizing handgrip strength to height may be a better way of assessing muscle weakness than using absolute values or normalizing to BMI [61]. Measuring hand grip strength is a simple, inexpensive, and non-invasive test that provides valuable information about muscle strength, functional capacity, and the risk of various negative health outcomes in older adults. A weak hand grip should not be ignored as it can be an early sign of deteriorating health and indicate the need for interventions such as strength training, nutritional support, and treatment of co-morbidities. Monitoring hand grip strength can help identify older adults at increased risk and enable timely interventions to improve their health and quality of life.

From all of the above, it is evident the need to research the interrelationships of eating patterns, quality of life, and diseases (using logical regression). The mentioned parameters and their significance in people who adhere to the Mediterranean diet were examined. The first data that should be highlighted is that the odds of a person accepting a change in diet will increase if they are faced with a disease (2+ chronic diseases [OR] = 1.21, [CI] = 1.11–1.32), this is in line with Romagnolo and Selmin [63] research suggesting that diet is the solution to which attention is turned when facing the burden of disease. From the results of the logistic regression, we also see that those individuals who rated their health as very good/excellent with the SPHUS ([OR] = 1.42, [CI] = 1.20–1.69) were significantly more likely to have an MD dietary pattern, and individuals with medium and high maximal handgrip were also strongly and consistently associated with a higher likelihood of adhering to the MD (medium: [OR] = 1.44, [CI] = 1.18–1.74; high: [OR] = 1.27, [CI] = 1.10–1.48). Although this paper uses a non-validated assessment of adherence to MD, these results are in accordance with research presented by Rippe [64], who emphasizes that it is dietary patterns, including MD, that have a significant impact on short-term and long-term health and quality of life. The same is confirmed by other studies [65,66,67] in which the connection with the handshake in the elderly population is emphasized.

There is increasing evidence that many conditions now increasing in prevalence in the middle-aged and older population actually need to be monitored and shared with the wider community to serve as a basis for further longitudinal research.

## 5. Conclusions

This study provides important insights into evolving dietary patterns and associated health outcomes among middle-aged and elderly populations in Europe based on data from the SHARE study. The analysis confirms that adherence to the Mediterranean dietary pattern is positively associated with better self-perceived health, lower prevalence of chronic diseases, and stronger handgrip strength—an essential biomarker of functional health in older adults. However, due to the limitations of the collected data in the SHARE database, the results are based on an unvalidated MD indicator. Simultaneously, it highlights the complex relationship between body mass index, physical inactivity, and nutritional status, particularly in populations over 65. The inclusion of grip strength as a functional measure, alongside nutritional indicators such as BMI and dietary adherence, enriches understanding of ageing-related health dynamics.

The findings underscore the importance of promoting healthy dietary habits and maintaining physical function through non-pharmacological strategies as part of public health policies targeting ageing populations. By leveraging multidimensional data from across Europe, this research supports the development of tailored interventions aimed at reducing obesity, preventing sarcopenia, and improving overall quality of life in older adults. Continued monitoring and cross-national comparisons using standardized data such as SHARE are crucial for guiding evidence-based health strategies in the context of demographic ageing.

## Figures and Tables

**Table 1:** Baseline characteristics and maximal grip strength of middle-aged and elderly Europeans following the Mediterranean dietary patterns.

Variables	Mediterranean Dietary Pattern (MD)			Maximum Grip Strength Measure			
	No (*n* = 201,967)	Yes (*n* = 56,386)	*p*-Value	Low (*n* = 75,439)	Medium (*n* = 163,149)	High (*n* = 19,635)	*p*-Value
Sex (%)			0.731				<0.001
Male	43.9	42.2		9.1	52.7	99.4	
Female	56.1	57.8		90.9	47.3	0.6	
Age (%)			0.997				<0.001
51–64	26.7	26.5		20.3	37.6	62.2	
65–74	34.9	35.7		32.2	38.3	32.0	
75–85	23.5	23.3		33.1	19.1	4.7	
>85	8.5	8.0		13.9	3.6	0.1	
BMI (%)			0.900				0.006
Normal (under 65 years)	33.1	33.7		36.1	32.9	20.9	
Normal (>65 years)	50.5	51.0		48.6	52.2	46.2	
Marital status (%)			0.636				0.128
Living with a partner	59.5	61.8		51.0	68.8	75.2	
Not living with a partner	40.4	38.1		48.9	31.2	24.6	
Education level (%)			0.929				0.028
Primary	32.4	33.7		41.7	25.0	15.4	
Secondary	35.6	33.9		33.5	40.0	43.5	
Tertiary	31.0	31.6		23.8	34.3	40.3	
2+ chronic diseases (%)			0.809				0.035
Less than 2 diseases	46.1	47.3		37.2	53.4	63.5	
2+ chronic diseases	53.8	52.6		62.7	46.6	36.4	
Physical inactivity (%)			0.901				0.003
Other	86.4	86.1		80.9	93.1	97.6	
Never vigorous nor moderate physical activity	13.4	13.8		19.1	6.9	2.4	
Self-perceived health (SPHUS-1) (%)			0.999				0.200
Excellent	5.8	5.7		3.5	7.2	9.8	
Very good	16.5	17.4		11.6	19.3	24.5	
Good	37.9	37.9		33.9	40.8	41.4	
Fair	28.8	28.4		36.0	26.4	19.6	
Poor	10.9	10.6		15.0	6.3	3.7	
SPHUS-2 (%)			0.867				0.064
Very good/excellent	22.3	23.0		15.0	26.4	35.3	
Less than very good	77.6	76.9		85.0	73.5	64.7	
CASP categories (%)			0.965				0.082
Low	2.7	2.9		5.6	1.8	0.8	
Medium	27.9	28.9		35.5	23.3	16.3	
High	69.4	68.2		60.3	75.3	83.2	
Current job situation (%)			0.737				<0.001
Retired	66.9	65.3		70.3	62.9	43.1	
Not retired	33.0	34.6		29.7	37.1	56.9	

Significance is indicated by *p* < 0.05.

**Table 2:** Distribution of the frequencies of body mass index (BMI), which is considered a nutritional status indicator. Two BMI categories are used based on age.

Sex	BMI Categories (kg/m^2^)	Age (Years)	BMI Categories * (kg/m^2^)	Age (Years)		
		51–64.9		65–74.9	75–85	>85
Male	<18.5—underweight	0.6	<18.5—severe underweight	2.6	3.2	7.4
			18.5–20.9—underweight	2.4	3.4	5.3
	18.5–24.9—normal	25.2	21–27.49—normal	49.4	55.8	59.0
	25–29.9—overweight	45.7	27.5–30.9—overweight	27.0	23.4	19.6
	>30—obese	26.5	31–39.9—obese	17.2	13.7	8.5
			>40—morbid obesity	1.4	0.6	0.1
Female	<18.5—underweight	1.2	<18.5—severe underweight	4.5	7.0	14.3
			18.5–20.9—underweight	6.4	6.4	9.4
	18.5–24.9—normal	37.0	21–27.49—normal	46.8	47.9	46.5
	25–29.9—overweight	34.8	27.5–30.9—overweight	21.4	20.6	16.9
	>30—obese	24.4	31–39.9—obese	19.0	16.8	12.5
			>40—morbid obesity	2.0	1.3	0.5

* ESPEN: BMI categories for adults over 65 years old [34].

**Table 3:** Logistic regression of different quality of life parameters of the elderly (the number of chronic diseases, nutritional status, physical inactivity, CASP, SPHUS, and maximal grip strength) and MD adherence.

	Crude Model		Model 1		Model 2	
	OR (95% CI)	*p*-Value	OR (95% CI)	*p*-Value	OR (95% CI)	*p*-Value
No. of chronic diseases						
Less than 2 diseases	Reference		Reference		Reference	
2+ chronic diseases	1.08 (1.03–1.12)	0.001	1.12 (1.09–1.30)	<0.001	1.21 (1.11–1.32)	<0.001
BMI						
Underweight	Reference		Reference		Reference	
Normal	1.16 (1.16–1.69)	0.001	1.28 (0.95–1.95)	0.090	1.39 (1.00–1.94)	0.048
Overweight	1.17 (0.99–1.40)	0.059	1.29 (0.65–1.77)	0.097	1.40 (1.00–1.94)	0.049
Obesity	1.07 (0.91–1.28)		1.17 (0.86–1.02)	0.317	1.33 (0.95–1.87)	0.094
CASP						
Low	Reference		Reference		Reference	
Medium	0.69 (0.89–0.81)	<0.001	0.75 (0.56–1.00)	0.049	0.92 (0.68–1.26)	0.616
High	0.93 (0.89–0.97)	0.001	0.94 (0.86–1.02)	0.131	0.95 (0.87–1.04)	0.240
Physical inactivity						
Never, nor moderate physical activity	Reference		Reference		Reference	
Other	0.79 (0.74–0.84)	<0.001	0.70 (0.61–0.79)	<0.001	0.63 (0.55–0.723)	<0.001
Self-perceived health (SPHUS-1)						
Poor	Reference		Reference		Reference	
Fair	0.96 (0.79–5.70)	0.133	0.79 (0.09–6.62)	0.825	0.84 (0.10–7.08)	0.872
Good	1.14 (1.00–1.26)	0.058	1.17 (0.94–1.44)	0.154	1.21 (0.96–1.52)	0.110
Very good	1.28 (1.15–1.39)	<0.001	1.35 (1.13–1.61)	0.001	1.44 (1.19–1.75)	<0.001
Excellent	1.24 (1.13–1.34)	<0.001	1.26 (1.08–1.48)	0.004	1.36 (1.14–1.62)	0.001
SPHUS-2						
Less than very good	Reference		Reference		Reference	
Very good/excellent	1.15 (1.08–1.26)	<0.001	1.29 (1.11–1.51)	0.001	1.42 (1.20–1.69)	<0.001
Maximal grip strength						
Low	Reference		Reference		Reference	
Medium	1.33 (1.22–1.45)	<0.001	1.38 (1.15–1.65)	0.001	1.36 (1.18–1.74)	<0.001
High	1.24 (1.14–1.34)	<0.001	1.23 (1.06–1.42)	0.005	1.27 (1.10–1.48)	0.002

Adjusted for age, sex, living with a partner, education, and current job status. Note: reported significant ORs where the confidence interval includes 1 do not imply statistical significance even though the *p*-values are less than 0.05 and/or 0.01.

## Data Availability

Data are available at: https://share-eric.eu/data/data-access, accessed on 15 November 2024.

## Supplementary Materials

The following supporting information can be downloaded at: https://www.mdpi.com/article/10.3390/nu17152525/s1, Table S1: Proportion of adults aged 51–65 years with different standard BMI categories (<18.5 kg/m^2^—underweight; 18.5–24.9 kg/m^2^—normal; 25–29.9 kg/m^2^—overweight and >30 kg/m^2^—obese) included in the SHARE study. Colour gradation from red (the lowest proportions), through white to blue (the highest proportion) within a wave; Table S2: Proportion of adults aged over 65 years in different ESPEN BMI categories (<20.9 kg/m^2^—underweight; 21–27.49 kg/m^2^—normal; 27.5–30.9 kg/m^2^—overweight and >31 kg/m^2^—obese) in the SHARE study. Colour gradation from red (the lowest proportion), through white to blue (the highest proportion) within a wave.

## Abbreviations

The following abbreviations are used in this manuscript:

BMI	Body mass index
CASP	Control, autonomy, self-realization, and pleasure scale
CVD	Cardiovascular diseases
CRM	Crude regression model
LDL/HDL	Low high-density lipoprotein cholesterol
MD	Mediterranean dietary pattern
OR	Odds ratios
SHARE	Survey of Health, Ageing and Retirement in Europe
SPHUS	Self-perceived health

## Appendix A

**Table A1:** Proportion of followers of the Mediterranean dietary pattern, by wave and country.

Country	Mediterranean Diet Pattern (%)							
	w6		w7		w8		w9	
	No	Yes	No	Yes	No	Yes	No	Yes
Austria	82.5	17.5	78.3	21.7	78.1	21.9	75.5	24.5
Germany	72.1	27.9	81.8	18.2	77.6	22.4	78.1	21.9
Sweden	75.8	24.2	78.3	21.7	77.4	22.6	81.0	19.0
Netherlands	n.a.	n.a.	0.0	0.0	75.9	24.1	77.0	23.0
Spain	63.3	36.7	62.2	37.8	60.9	39.1	56.4	43.6
Italy	77.2	22.8	74.9	25.1	73.5	26.5	73.8	26.2
France	84.5	15.5	86.8	13.2	85.5	14.5	84.9	15.1
Denmark	80.4	19.6	88.3	11.7	85.7	14.3	83.4	16.6
Greece	81.2	18.8	75.6	24.4	81.5	18.5	79.5	20.5
Switzerland	73.6	26.4	75.2	24.8	75.5	24.5	75.2	24.8
Belgium	84.5	15.5	91.6	8.4	91.2	8.8	89.2	10.8
Israel	73.5	26.5	0.0	0.0	71.6	28.4	70.4	29.6
Czech Republic	88.1	11.9	77.5	22.5	86.6	13.4	83.7	16.3
Poland	74.6	25.4	78.0	22.0	79.7	20.3	67.3	32.7
Luxembourg	67.2	32.8	0.0	0.0	82.1	17.9	84.2	15.8
Hungary	n.a.	n.a.	0.0	0.0	65.8	34.2	64.7	35.3
Portugal	74.4	25.6	0.0	0.0	0.0	0.0	87.9	12.1
Slovenia	70.9	29.1	0.0	0.0	71.2	28.8	71.1	28.9
Estonia	87.6	12.4	0.0	0.0	85.7	14.3	86.1	13.9
Croatia	70.2	29.8	0.0	0.0	82.4	17.6	85.6	14.4
Lithuania	n.a.	n.a.	0.0	0.0	75.3	24.7	82.8	17.2
Bulgaria	n.a.	n.a.	0.0	0.0	79.7	20.3	75.7	24.3
Cyprus	n.a.	n.a.	0.0	0.0	55.8	44.2	59.8	40.2
Finland	n.a.	n.a.	0.0	0.0	85.5	14.5	86.3	13.7
Latvia	n.a.	n.a.	0.0	0.0	74.7	25.3	79.3	20.7
Malta	n.a.	n.a.	0.0	0.0	54.5	45.5	57.1	42.9
Romania	n.a.	n.a.	0.0	0.0	65.1	34.9	50.8	49.2
Slovakia	n.a.	n.a.	0.0	0.0	84.1	15.9	81.7	18.3
Total	77.9	22.1	79.1	20.9	78.1	21.9	77.6	22.4

n.a. not available; w6, data collected in 2015; w7, data collected in 2017; w8, data collected in 2019/20; w9, data collected in 2021/22.

**Table A2:** The proportion of participants who achieved maximum grip, which was divided into three categories, for the four waves of data in the SHARE database.

Country	Max Grip in Three Categories (%)											
	w6			w7			w8			w9		
	L	M	H	L	M	H	L	M	H	L	M	H
Austria	28.4	63.0	8.6	29.5	63.6	6.9	37.2	56.8	5.8	26.3	66.5	7.2
Germany	19.8	68.1	12.1	21.4	67.8	10.8	24.9	67.3	7.6	18.9	72.4	8.6
Sweden	25.0	64.1	10.9	27.3	63.5	9.3	30.2	62.5	7.3	23.6	68.7	7.7
Netherlands	n.a.	n.a.	n.a.	0.0	0.0	0.0	22.9	68.1	8.9	20.7	72.0	7.2
Spain	42.8	54.3	2.9	45.2	52.1	2.7	49.1	48.3	2.6	39.6	58.1	2.2
Italy	29.9	63.3	6.8	31.6	63.7	4.6	38.2	58.2	3.4	31.4	65.8	2.6
France	30.6	62.3	7.1	31.0	61.8	7.3	33.2	60.1	6.7	26.9	64.9	8.2
Denmark	18.4	64.3	17.3	18.9	65.4	15.6	21.2	65.8	13.0	19.0	68.7	12.3
Greece	33.9	59.1	6.9	35.1	60.2	4.8	35.8	60.7	3.5	29.2	67.3	3.4
Switzerland	24.0	67.0	8.9	26.2	65.4	8.3	29.5	62.9	7.6	25.0	68.0	6.9
Belgium	25.4	63.7	10.9	26.1	64.3	9.6	29.5	61.0	9.5	24.2	66.3	9.4
Israel	49.8	47.9	2.1	45.8	52.0	1.9	46.3	52.1	1.3	36.4	61.9	1.5
Czech Republic	24.9	67.2	7.9	28.1	65.2	6.6	28.2	64.8	6.9	26.7	67.9	5.3
Poland	28.3	63.1	8.6	28.3	63.1	8.6	28.3	65.3	6.3	24.0	71.0	4.9
Luxembourg	23.9	65.9	10.3	25.5	64.8	9.7	25.3	67.1	7.5	19.7	73.3	6.7
Hungary	n.a.	n.a.	n.a.	38.1	56.6	5.3	39.0	55.2	5.4	33.7	61.4	5.0
Portugal	43.8	53.9	2.3	42.1	56.4	1.5	0.0	0.0	0.0	33.5	63.0	2.9
Slovenia	26.6	62.2	11.2	27.3	63.3	9.4	28.9	62.6	8.4	20.8	68.6	10.4
Estonia	30.1	60.2	9.6	30.5	61.0	8.4	32.1	59.9	7.9	24.9	65.6	9.4
Croatia	26.6	62.1	11.2	24.9	62.6	12.5	27.5	62.2	10.1	22.5	67.8	9.6
Lithuania	n.a.	n.a.	n.a.	26.6	65.0	8.4	28.3	63.4	8.3	28.3	66.5	5.1
Bulgaria	n.a.	n.a.	n.a.	41.7	55.2	3.1	44.7	54.7	0.6	35.3	60.4	4.2
Cyprus	n.a.	n.a.	n.a.	50.2	46.7	3.1	58.6	40.3	0.6	40.8	58.2	0.9
Finland	n.a.	n.a.	n.a.	20.5	66.6	12.9	23.6	67.6	8.9	20.4	70.6	9.0
Latvia	n.a.	n.a.	n.a.	26.1	61.6	12.4	25.0	65.9	9.2	21.5	70.8	7.7
Malta	n.a.	n.a.	n.a.	42.8	55.1	2.0	43.5	54.3	2.1	34.4	63.7	1.8
Romania	n.a.	n.a.	n.a.	33.7	61.5	4.7	34.3	62.0	3.8	26.6	70.6	2.8
Slovakia	n.a.	n.a.	n.a.	25.4	62.7	11.9	44.4	47.9	7.6	32.5	64.8	2.6
Total	28.8	62.2	8.9	30.4	61.8	7.8	32.0	61.2	6.8	25.6	67.4	6.9

L: Low; M: Medium; H: High; n.a. not available; w6, data collected in 2015; w7, data collected in 2017; w8, data collected in 2019/20; w9, data collected in 2021/22.

**Table A3:** The most common Mediterranean diet scores used in research.

Score (Abbreviation)	Components that Are Scored	Score Range	Cut-Offs *	Source (Reference)
Mediterranean Diet Score (MDS)	Vegetables, legumes, fruits/nuts, cereals, fish, meat, dairy, alcohol, MUFA/SFA ratio	0–9	Low: 0–3 Moderate: 4–5 High: 6–9	Trichopoulou, et al. [51]
Alternate Mediterranean Diet Score (aMED)	Vegetables, legumes, fruits, nuts, whole grains, fish, red/processed meats, alcohol, fats	0–9	Low: 0–3 Moderate: 4–5 High: 6–9	Fung, et al. [52]
Mediterranean Diet Adherence Screener (MEDAS)	Olive oil, vegetables, fruits, red meats, butter/cream, sugary drinks, legumes, wine, etc.	0–14	Low: 0–5 Moderate: 6–9 High: ≥10	Martínez-González, et al. [50]
Mediterranean-Style Dietary Pattern Score (MSDPS)	13 food groups: whole grains, fruits, vegetables, dairy, olive oil, fish, legumes, alcohol	0–100	Low: <50 Moderate: 50–75 High: >75	Rumawas, et al. [53]
Mediterranean Diet Quality Index for Children and Adolescents (KIDMED)	Fruits, vegetables, fast food, sweets, breakfast habits, dairy, cereals, nuts	−4 to +12	Low: ≤3 Moderate: 4–7 High: ≥8	Serra-Majem, et al. [54]

* Most commonly used cut-offs.

## References

[1] Eurostat (2025). Ageing Europe-Statistics on Population Developments. https://ec.europa.eu/eurostat/statistics-explained/index.php?title=Ageing_Europe_-_statistics_on_population_developments.

[2] Arvanitis M., Lowenstein C.J. (2023). Dyslipidemia. Ann. Intern. Med..

[3] OECD (2023). Health at A Glance 2023: OECD Indicators.

[4] Yan Y., Wu T., Zhang M., Li C., Liu Q., Li F. (2022). Prevalence, Awareness and Control of Type 2 Diabetes Mellitus and Risk Factors in Chinese Elderly Population. BMC Public Health.

[5] Bilal A., Pratley R. (2025). Diabetes and cardiovascular disease in older adults. Ann. N. Y. Acad. Sci..

[6] Mensah G., Fuster V., Roth G. (2023). A Heart-Healthy and Stroke-Free World: Using Data to Inform Global Action. JACC.

[7] Jebari-Benslaiman S., Galicia-García U., Larrea-Sebal A., Olaetxea J.R., Alloza I., Vandenbroeck K., Benito-Vicente A., Martín C. (2022). Pathophysiology of Atherosclerosis. Int. J. Mol. Sci..

[8] Riccardi G., Giosuè A., Calabrese I., Vaccaro O. (2022). Dietary recommendations for prevention of atherosclerosis. Cardiovasc. Res..

[9] Lusis A.J., Björkegren J.L.M. (2022). Atherosclerosis: Recent Developments. Cell.

[10] Ajoolabady A., Pratico D., Lin L., Mantzoros C.S., Bahijri S., Tuomilehto J., Ren J. (2024). Inflammation in atherosclerosis: Pathophysiology and mechanisms. Cell Death Dis..

[11] Gaur A., Carr F., Warriner D. (2024). Cardiogeriatrics: The current state of the art. Heart.

[12] Sciacqua A., Succurro E., Armentaro G., Miceli S., Pastori D., Rengo G., Sesti G. (2023). Pharmacological treatment of type 2 diabetes in elderly patients with heart failure: Randomized trials and beyond. Heart Fail. Rev..

[13] Grajower M.M., LeRoith D. (2023). Management of Type 2 Diabetes Mellitus in the Very Elderly: One Practice’s Experience. Endocr. Pract..

[14] Gadó K., Tabák G.Á., Vingender I., Domján G., Dörnyei G. (2024). Treatment of type 2 diabetes mellitus in the elderly-Special considerations. Physiol. Int..

[15] American Diabetes Association Professional Practice Committee (2025). 13. Older Adults: Standards of Care in Diabetes—2025. Diabetes Care.

[16] Song R., Hu M., Qin X., Qiu L., Wang P., Zhang X., Liu R., Wang X. (2023). The Roles of Lipid Metabolism in the Pathogenesis of Chronic Diseases in the Elderly. Nutrients.

[17] Katsiki N., Kolovou G., Perez-Martinez P., Mikhailidis D.P. (2018). Dyslipidaemia in the Elderly: To Treat or Not to Treat?. Expert Rev. Clin. Pharmacol..

[18] Gielen E., Dupont J., Dejaeger M., Laurent M.R. (2023). Sarcopenia, osteoporosis and frailty. Metab. Clin. Exp..

[19] Xu J., Wan C.S., Ktoris K., Reijnierse E.M., Maier A.B. (2022). Sarcopenia is Associated with Mortality in Adults: A Systematic Review and Meta-Analysis. Gerontology.

[20] Shu X., Lin T., Wang H., Zhao Y., Jiang T., Peng X., Yue J. (2022). Diagnosis, prevalence, and mortality of sarcopenia in dialysis patients: A systematic review and meta-analysis. J. Cachexia Sarcopenia Muscle.

[21] Bohannon R.W. (2019). Grip Strength: An Indispensable Biomarker for Older Adults. Clin. Interv. Aging.

[22] Zhao X., An X., Yang C., Sun W., Ji H., Lian F. (2023). The crucial role and mechanism of insulin resistance in metabolic disease. Front. Endocrinol..

[23] Mancia G., Kreutz R., Brunström M., Burnier M., Grassi G., Januszewicz A., Muiesan M.L., Tsioufis K., Agabiti-Rosei E., Algharably E.A.E. (2023). 2023 ESH Guidelines for the Management of Arterial Hypertension: The Task Force for the Management of Arterial Hypertension of the European Society of Hypertension. J. Hypertens..

[24] Visseren F.L., Mach F., Smulders Y.M., Carballo D., Koskinas K.C., Bäck M., Benetos A., Biffi A., Boavida J.-M., Capodanno D. (2021). 2021 ESC guidelines on Cardiovascular Disease Prevention in Clinical Practice. Eur. Heart J..

[25] Obeidat A.A., Ahmad M.N., Ghabashi M.A., Alazzeh A.Y., Habib S.M., Abu Al-Haijaa D., Azzeh F.S. (2025). Developmental Trends of Metabolic Syndrome in the Past Two Decades: A Narrative Review. J. Clin. Med..

[26] Santos C.A., Maia H.F., Pitanga F.J.G., de Almeida M.D.C.C., da Fonseca M.J.M., de Aquino E.M.L., Cardoso L.O., Griep R.H., Barreto S.M., Suemoto C.K. (2025). Hand Grip Strength Cut-Off Points as a Discriminator of Sarcopenia and Sarcopenic Obesity: Results from the ELSA-Brasil Cohort. J. Cachexia Sarcopenia Muscle.

[27] Furbatto M., Lelli D., Antonelli Incalzi R., Pedone C. (2024). Mediterranean Diet in Older Adults: Cardiovascular Outcomes and Mortality from Observational and Interventional Studies—A Systematic Review and Meta-Analysis. Nutrients.

[28] Scaglione S., Di Chiara T., Daidone M., Tuttolomondo A. (2025). Effects of the Mediterranean Diet on the Components of Metabolic Syndrome Concerning the Cardiometabolic Risk. Nutrients.

[29] Börsch-Supan A., Brandt M., Hunkler C., Kneip T., Korbmacher J., Malter F., Schaan B., Stuck S., Zuber S., SHARE Central Coordination Team (2013). Data Resource Profile: The Survey of Health, Ageing and Retirement in Europe (SHARE). Int. J. Epidemiol..

[30] Bergmann M., Scherpenzeel A., Börsch-Supan A. (2019). SHARE Wave 7 Methodology: Panel Innovations and Life Histories.

[31] Börsch-Supan A. (2022). Survey of Health, Ageing and Retirement in Europe (SHARE) Wave 8.

[32] Maltarić M., Ruščić P., Kolak M., Bender D.V., Kolarić B., Ćorić T., Hoejskov P.S., Bošnir J., Kljusurić J.G. (2023). Adherence to the Mediterranean Diet Related to the Health Related and Well-Being Outcomes of European Mature Adults and Elderly, with an Additional Reference to Croatia. Int. J. Environ. Res. Public Health.

[33] Alves R., Perelman J. (2022). European mature adults and elderly are moving closer to the Mediterranean diet—A longitudinal study, 2013–2019. Eur. J. Public Health.

[34] Alves R. (2021). Mediterranean Diet in Europe: How are mature adults and elderly moving closer to this diet pattern?. Eur. J. Public Health.

[35] Lee M.T., Howe T.H., Chen C.C., Wu C.Y., Hsieh Y.W. (2023). Grip strength differences in middle-aged and older adults and individuals with stroke. Eur. J. Phys. Rehabil. Med..

[36] CDC (2024). Adult BMI Categories. https://www.cdc.gov/bmi/adult-calculator/bmi-categories.html.

[37] Volkert D., Beck A.M., Cederholm T., Cruz-Jentoft A., Hooper L., Kiesswetter E., Maggio M., Raynaud-Simon A., Sieber C., Sobotka L. (2022). ESPEN practical guideline: Clinical nutrition and hydration in geriatrics. Clin. Nutr..

[38] SHARE (2024). Release Guide 9.0.0. https://share-eric.eu/fileadmin/user_upload/Release_Guides/SHARE_release_guide_9-0-0.pdf.

[39] Heymans M.W., Twisk J.W.R. (2022). Handling missing data in clinical research. J. Clin. Epidemiol..

[40] IEssien U.A., Amechi K.U., Madu K.A., Ede O., Iyidobi E.C., Anyaehie U.E., Obadaseraye O.R., Ogbonnaya I.S., Ogbu D.C., Ngwangwa C.L. (2023). Assessment of handgrip strength in healthy african subjects: Establishing age and gender stratified reference values. Niger. J. Clin. Pract..

[41] López-Bueno R., Calatayud J., Andersen L.L., Casaña J., Koyanagi A., Cruz B.d.P., Smith L. (2023). Dose–response association of handgrip strength and risk of depression: A longitudinal study of 115 601 older adults from 24 countries. Br. J. Psychiatry.

[42] Lin H., Ren H. (2021). The Influence of Interpersonal Behaviors and Population Density on Grip Strength of Elderly People: An Analysis of the Direct vs. Indirect Effects via Social Participation. Front. Public Health.

[43] Ghosh T.S., Rampelli S., Jeffery I.B., Santoro A., Neto M., Capri M., Giampieri E., Jennings A., Candela M., Turroni S. (2020). Mediterranean diet intervention alters the gut microbiome in older people reducing frailty and improving health status: The NU-AGE 1-year dietary intervention across five European countries. Gut.

[44] Chaaya C., Raad E., Kahale F., Chelala E., Ziade N., Maalouly G. (2025). Adherence to Mediterranean Diet and Ocular Dryness Severity in Sjögren’s Syndrome: A Cross-Sectional Study. Med. Sci..

[45] Castelló J.V., Tubianosa C. (2020). Linking Mediterranean Diet and Lifestyle with Cardio Metabolic Disease and Depressive Symptoms: A Study on the Elderly in Europe. Int. J. Environ. Res. Public Health.

[46] Mazza E., Ferro Y., Maurotti S., Micale F., Boragina G., Russo R., Lascala L., Sciacqua A., Gazzaruso C., Montalcini T. (2024). Association of dietary patterns with sarcopenia in adults aged 50 years and older. Eur. J. Nutr..

[47] Cacciatore S., Gava G., Calvani R., Marzetti E., Coelho-Junior H.J., Picca A., Esposito I., Ciciarello F., Salini S., Russo A. (2023). Lower Adherence to A Mediterranean Diet is Associated with High Adiposity in Community-Dwelling Older Adults: Results from the Longevity Check-Up (Lookup) 7+ Project. Nutrients.

[48] Marcos-Pardo P.J., González-Gálvez N., López-Vivancos A., Espeso-García A., Martínez-Aranda L.M., Gea-García G.M., Orquín-Castrillón F.J., Carbonell-Baeza A., Jiménez-García J.D., Velázquez-Díaz D. (2020). Sarcopenia, Diet, Physical Activity and Obesity in European Middle-Aged and Older Adults: The LifeAge Study. Nutrients.

[49] Coelho-Júnior H.J., Trichopoulou A., Panza F. (2023). Protein Intake and Physical Activity in Relation to Grip Strength Decline: A Pooled Analysis of Four Longitudinal Ageing Cohorts. Clin. Nutr..

[50] Martínez-González M.A., García-Arellano A., Toledo E., Salas-Salvadó J., Buil-Cosiales P., Corella D., Covas M.I., Schröder H., Arós F., Gómez-Gracia E. (2012). A 14-Item Mediterranean Diet Assessment Tool and Obesity Indexes among High-Risk Subjects: The PREDIMED Trial. PLoS ONE.

[51] Trichopoulou A., Costacou T., Bamia C., Trichopoulos D. (2003). Adherence to a Mediterranean Diet and Survival in a Greek Population. N. Engl. J. Med..

[52] Fung T.T., McCullough M.L., Newby P.K., Manson J.E., Meigs J.B., Rifai N., Willett W.C., Hu F.B. (2005). Diet-Quality Scores and Plasma Concentrations of Markers of Inflammation and Endothelial Dysfunction. Am. J. Clin. Nutr..

[53] Rumawas M.E., Dwyer J.T., McKeown N.M., Meigs J.B., Rogers G., Jacques P.F. (2009). The Development of the Mediterranean-Style Dietary Pattern Score and Its Application to the American Diet in the Framingham Offspring Cohort. J. Nutr..

[54] Serra-Majem L., Ribas L., Ngo J., Ortega R.M., García A., Pérez-Rodrigo C., Aranceta J. (2004). Food, Youth and the Mediterranean Diet in Spain. Development of KIDMED, Mediterranean Diet Quality Index in Children and Adolescents. Public Health Nutr..

[55] Zhang S., Gu Y., Rayamajhi S., Thapa A., Meng G., Zhang Q., Liu L., Wu H., Zhang T., Wang X. (2022). Ultra-processed food intake is associated with grip strength decline in middle-aged and older adults: A prospective analysis of the TCLSIH study. Eur. J. Nutr..

[56] Ma Z., Yang H., Meng G., Zhang Q., Liu L., Wu H., Gu Y., Zhang S., Wang X., Zhang J. (2023). Anti-inflammatory dietary pattern is associated with handgrip strength decline: A prospective cohort study. Eur. J. Nutr..

[57] Follis S., Cook A., Bea J.W., Going S.B., Laddu D., Cauley J.A., Shadyab A.H., Stefanick M.L., Chen Z. (2018). Association Between Sarcopenic Obesity and Falls in a Multiethnic Cohort of Postmenopausal Women. J. Am. Geriatr. Soc..

[58] Giovannini S., Brau F., Galluzzo V., Santagada D.A., Loreti C., Biscotti L., Laudisio A., Zuccalà G., Bernabei R. (2022). Falls among Older Adults: Screening, Identification, Rehabilitation and Management. Appl. Sci..

[59] Aballay L.R., Eynard A.R., del Pilar Díaz M., Navarro A., Muñoz S.E. (2013). Overweight and obesity: A review of their relationship to metabolic syndrome, cardiovascular disease, and cancer in South America. Nutr. Rev..

[60] Huemer M.-T., Kluttig A., Fischer B., Ahrens W., Castell S., Ebert N., Gastell S., Jöckel K.-H., Kaaks R., Karch A. (2023). Grip strength values and cut-off points based on over 200,000 adults of the German National Cohort-a comparison to the EWGSOP2 cut-off points. Age Ageing.

[61] Huang L., Shen X., Zou Y., Wang Y. (2024). Effects of BMI and grip strength on older adults’ falls-A longitudinal study based on CHARLS. Front. Public Health.

[62] Liu S., He Y., Yu G., Song C., Wang D., Liu L., Liang H., Wan H., Shen J. (2024). Association of muscle mass, grip strength and fat-to-muscle ratio and metabolic dysfunction-associated steatotic liver disease in a middle-to-elderly aged population. Ann. Med..

[63] Romagnolo D.F., Selmin O.I. (2017). Mediterranean Diet and Prevention of Chronic Diseases. Nutr. Today.

[64] Sarpdaği Y., Yiğit M.F., Aydin M.A., Yildirim M.S., Çiftci N., Yildiz M. (2025). Mediating role of psychological well-being in the effect of spirituality on attitudes toward death in the elderly. Psychogeriatr. Off. J. Jpn. Psychogeriatr. Soc..

[65] Pallazola V.A., Davis D.M., Whelton S.P., Cardoso R., Latina J.M., Michos E.D., Sarkar S., Blumenthal R.S., Arnett D.K., Stone N.J. (2019). A Clinician’s Guide to Healthy Eating for Cardiovascular Disease Prevention. Mayo Clin. Proc. Innov. Qual. Outcomes.

[66] Rippe J.M. (2018). Lifestyle Medicine: The Health Promoting Power of Daily Habits and Practices. Am. J. Lifestyle Med..

[67] Jurek J.M., Zablocka-Sowinska K., Clavero Mestres H., Reyes Gutiérrez L., Camaron J., Auguet T. (2025). The Impact of Dietary Interventions on Metabolic Outcomes in Metabolic Dysfunction-Associated Steatotic Liver Disease (MASLD) and Comorbid Conditions, Including Obesity and Type 2 Diabetes. Nutrients.

