# Supplementary material for: Dietary Habits and Obesity in Middle-Aged and Elderly Europeans—The Survey of Health, Ageing, and Retirement in Europe (SHARE)
Source: Nutrients. 2025 Jul 31;17(15):2525. doi: 10.3390/nu17152525 (PMC12348933; doi:10.3390/nu17152525)
Supplement: Supplementary file 1 [file nutrients-17-02525-s001.zip › nutrients-3646610-supplementary.pdf]

Table S1. Share of the BMI (kg/m^2) of the adults included in the SHARE study, aged 51-65 years, regarding the standard BMI categories (<18.5 kg/m^2–underweight; 18.5–24.9 kg/m^2–normal; 25–29.9 kg/m^2–overweight and >30 kg/m^2–obese). The color gradation: from red (the lowest values), through white to blue (the highest shares) within a wave.

Country	W6				W7				W8				W9			
	underweight	normal	overweight	obese	underweight	**normal**	overweight	obese	underweight	normal	overweight	obese	underweight	normal	overweight	obese
Austria	1.3	**36.2**	39.5	22.5	2.1	**41.1**	32.2	21.7	1.3	**35.5**	40.1	21.4	1.2	**37.7**	36.3	22.7
Germany	0.8	**34.9**	39.7	23.9	1.3	**39.5**	33.5	23.6	0.7	**35.4**	38.1	25.0	0.7	**36.0**	37.6	24.3
Sweden	0.7	**37.5**	41.1	19.3	1.9	**46.0**	32.6	15.7	0.3	**39.7**	40.3	17.5	0.0	**39.2**	38.7	20.4
Netherlands	/	/	/	/	/	/	/	/	1.2	**41.7**	36.5	19.6	1.7	**42.4**	34.9	19.7
Spain	0.8	**34.1**	42.7	19.0	1.8	**32.2**	35.1	18.5	1.0	**32.0**	40.8	23.7	1.3	**32.1**	44.9	18.4
Italy	0.9	**45.5**	38.6	14.1	2.4	**44.6**	35.3	15.7	1.0	**39.1**	41.4	15.2	1.1	**40.8**	41.9	14.4
France	1.8	**39.1**	35.9	21.8	3.1	**43.7**	31.1	18.8	2.0	**42.5**	34.3	20.0	2.0	**39.5**	36.0	20.7
Denmark	1.1	**41.0**	39.0	17.4	2.8	**45.7**	31.6	16.3	1.2	**39.0**	35.0	22.9	0.5	**36.3**	36.6	25.2
Greece	0.6	**32.0**	45.8	21.0	0.2	**35.0**	42.4	20.0	0.3	**32.2**	47.9	18.9	0.7	**34.6**	47.8	16.4
Switzerland	1.6	**47.7**	34.9	15.4	3.8	**51.2**	30.0	13.1	1.8	**48.1**	31.3	18.4	2.7	**43.7**	35.8	16.8
Belgium	1.7	**37.6**	37.6	21.8	3.0	**41.5**	32.2	19.3	2.0	**40.2**	37.0	19.6	1.7	**36.9**	37.8	22.4
Israel	1.7	**32.9**	42.3	18.9	1.1	**33.6**	32.2	18.1	0.8	**35.5**	38.7	8.9	1.4	**33.8**	39.2	17.6
Czech Republic	0.4	**27.0**	37.8	32.6	0.8	**25.0**	38.6	30.8	0.6	**26.8**	36.6	34.2	1.0	**29.2**	38.7	29.8
Poland	1.2	**33.5**	38.0	26.4	1.0	**30.5**	37.1	28.2	1.1	**26.9**	39.0	31.3	0.7	**29.6**	39.4	27.7
Luxembourg	1.7	**37.5**	35.7	23.6	2.4	**40.4**	32.7	20.0	0.8	**35.2**	39.4	20.7	2.3	**32.4**	37.4	23.3
Hungary	/	/	/	/	1.3	**30.2**	36.5	29.0	0.6	**27.0**	34.4	36.2	0.5	**21.7**	46.1	30.8
Portugal	0.6	**29.0**	42.1	25.2	1.5	**30.7**	36.2	22.0	/	/	/	/	0.9	**27.2**	42.5	24.1
Slovenia	0.5	**29.3**	43.3	26.0	1.1	**31.2**	41.1	22.7	0.6	**28.8**	43.2	26.1	0.6	**29.2**	42.1	25.9
Estonia	1.0	**30.2**	36.9	31.0	1.2	**26.7**	35.2	33.4	1.2	**25.9**	35.5	36.6	1.0	**29.8**	35.5	32.6
Croatia	0.6	**31.2**	43.6	23.1	0.7	**29.2**	41.8	25.7	0.2	**30.1**	42.2	26.2	0.7	**28.3**	39.7	28.4
Lithuania	/	/	/	/	1.0	**26.3**	37.2	34.2	0.3	**26.5**	40.6	32.1	0.6	**26.3**	40.9	30.7
Bulgaria	/	/	/	/	1.8	**32.0**	39.1	24.9	0.7	**30.0**	38.0	24.2	0.8	**20.2**	35.3	33.3
Cyprus	/	/	/	/	1.0	**29.2**	34.0	20.9	0.0	**35.8**	41.5	19.8	1.1	**35.6**	40.6	20.0
Finland	/	/	/	/	0.8	**35.6**	37.7	22.8	0.7	**30.9**	39.2	27.0	0.4	**29.0**	39.9	27.8
Latvia	/	/	/	/	1.0	**24.7**	36.6	34.5	1.0	**25.0**	31.4	40.2	0.6	**26.9**	42.0	28.6
Malta	/	/	/	/	0.7	**22.8**	30.9	30.2	0.4	**14.5**	31.2	41.3	0.5	**15.5**	29.2	35.2
Romania	/	/	/	/	1.8	**27.5**	36.4	32.2	0.2	**25.3**	38.1	34.8	0.4	**21.4**	35.9	37.1
Slovakia	/	/	/	/	2.1	**36.2**	37.2	24.1	0.7	**29.6**	44.4	22.7	0.7	**31.9**	42.7	22.5
Total	1.0	**35.4**	39.7	22.5	1.7	**35.0**	35.5	23.4	0.9	**32.8**	38.8	25.4	0.9	**32.1**	39.3	25.3

Table S2. Share of the BMI (kg/m^2) of the adults included in the SHARE study, over 65 years, regarding the ESPEN BMI categories (<20.9 kg/m^2–underweight; 21–27.49 kg/m^2–normal; 27.5–30.9 kg/m^2–overweight and >31 kg/m^2–obese). The color gradation: from red (the lowest values), through white to blue (the highest shares) within a wave.

Country	W6				W7				W8				W9			
	underweight	normal	overweight	obese	underweight	normal	overweight	obese	underweight	normal	overweight	obese	underweight	normal	overweight	obese
Austria	7.5	**53.0**	21.0	17.5	9.5	**52.7**	19.0	16.3	9.3	**54.9**	21.0	14.8	10.8	**53.4**	20.9	14.9
Germany	5.8	**53.1**	22.6	17.8	6.1	**49.4**	23.0	20.3	6.7	**55.6**	21.2	16.5	8.2	**53.7**	20.3	17.8
Sweden	7.4	**59.4**	19.3	12.1	6.7	**55.8**	22.2	13.3	8.4	**61.6**	16.8	13.2	10.4	**59.9**	17.6	12.0
Netherlands	/	/	/	/	/	/	/	/	8.5	**60.3**	18.6	12.5	7.9	**61.1**	19.4	11.7
Spain	4.4	**52.0**	22.1	13.8	6.2	**53.6**	21.2	14.8	5.2	**46.9**	22.3	25.6	18.9	**42.6**	22.1	16.4
Italy	8.0	**58.2**	20.7	11.9	9.0	**60.2**	18.1	11.4	7.2	**57.1**	21.3	14.4	10.5	**57.6**	21.0	10.8
France	10.6	**51.2**	19.5	16.4	12.7	**50.3**	20.7	15.0	11.2	**51.6**	21.0	16.2	13.6	**52.3**	19.8	14.3
Denmark	9.4	**57.2**	19.2	12.3	7.2	**54.6**	22.2	14.4	10.1	**56.8**	20.1	13.0	12.8	**56.6**	18.5	12.2
Greece	3.8	**52.7**	24.9	17.0	4.2	**54.2**	24.8	15.5	2.2	**53.3**	26.9	17.5	5.3	**54.6**	25.5	14.6
Switzerland	12.2	**58.1**	17.9	10.8	14.0	**56.7**	16.9	11.2	13.5	**57.9**	18.0	10.6	15.9	**58.0**	16.8	9.3
Belgium	9.7	**52.3**	19.7	15.6	9.4	**52.9**	18.7	17.7	9.7	**52.2**	21.2	16.8	12.3	**52.0**	20.4	15.3
Israel	5.5	**50.0**	22.1	16.2	6.7	**56.7**	14.0	11.5	6.0	**52.2**	19.0	22.8	15.8	**48.7**	19.6	15.8
Czech Republic	4.1	**42.3**	26.3	25.2	4.4	**40.9**	21.6	27.5	4.2	**43.9**	27.5	24.4	5.8	**44.0**	26.2	24.0
Poland	6.0	**45.3**	24.4	21.6	6.2	**47.4**	24.7	20.6	5.0	**43.6**	25.6	25.8	8.3	**44.0**	25.9	21.7
Luxembourg	8.1	**50.9**	19.9	19.3	8.1	**49.4**	20.6	18.9	8.8	**49.7**	19.8	21.7	10.5	**52.3**	19.5	17.7
Hungary	/	/	/	/	4.1	**40.5**	23.9	28.8	5.5	**47.6**	24.3	22.6	8.0	**42.9**	26.7	22.3
Portugal	4.8	**51.7**	23.1	15.9	5.0	**45.9**	24.6	18.2	/	/	/	/	12.4	**49.5**	23.3	14.8
Slovenia	4.2	**48.3**	26.2	19.4	5.8	**48.1**	25.4	18.8	5.0	**48.1**	26.2	20.8	9.5	**46.8**	24.7	18.9
Estonia	6.0	**45.3**	23.2	23.7	6.2	**43.5**	22.9	25.4	5.7	**44.9**	25.5	23.9	9.2	**43.1**	24.4	23.3
Croatia	4.7	**50.1**	25.0	18.0	3.7	**48.9**	27.1	19.1	4.3	**44.9**	27.6	23.3	9.4	**46.2**	24.6	19.8
Lithuania	/	/	/	/	4.5	**43.1**	27.0	25.4	5.6	**46.8**	23.4	24.2	9.6	**42.9**	22.7	24.9
Bulgaria	/	/	/	/	3.8	**48.6**	24.3	21.5	4.4	**45.4**	23.1	27.2	15.3	**41.6**	23.7	19.5
Cyprus	/	/	/	/	4.5	**49.1**	21.4	20.4	4.5	**43.3**	22.6	29.6	18.1	**45.7**	20.1	16.1
Finland	/	/	/	/	5.5	**52.6**	21.1	19.1	5.0	**55.8**	22.7	16.5	7.8	**53.3**	23.4	15.5
Latvia	/	/	/	/	4.4	**41.3**	26.5	26.1	3.4	**43.4**	25.9	27.4	5.3	**42.1**	25.7	27.0
Malta	/	/	/	/	3.4	**35.9**	20.1	32.3	1.4	**48.2**	22.7	27.7	26.3	**26.8**	17.3	29.7
Romania	/	/	/	/	5.1	**41.4**	27.0	25.6	5.6	**39.9**	23.7	30.8	13.1	**38.2**	24.3	24.5
Slovakia	/	/	/	/	5.9	**50.2**	27.5	16.2	2.1	**49.5**	24.5	23.8	7.1	**44.4**	26.6	21.9
Total	6.7	**51.8**	22.1	16.9	6.6	**49.5**	22.6	19.1	6.7	**50.9**	22.6	19.8	10.4	**49.6**	22.4	17.6
